# Metropolitan Hospital Sunday Fund

**Published:** 1895-11-16

**Authors:** 


### METROPOLITAN HOSPITAL SUNDAY FUND.

The Council of the Metropolitan Hospital Sunday Fund met in the saloon of the London Mansion House on Thursday, November 7th, to receive a second report from the Distribution Committee of the Fund. Sir Joseph Renals, Bart., the ex-Lord Mayor, presided. Amongst those present were: Sir Sydney H. Waterlow, Bart., his Eminence Cardinal Vaughan, Chief Rabbi Dr. H. Adler, the Ven. W. M. Sinclair, D.D. (Archdeacon of London), the Rev. Canon Fleming, the Rev. Dr. Donald McLeod, the Rev. S. B. Burnaby, the Rev. Albert A. Harland, the Rev. W. H. Harwood, the Rev. E. E. Jenkins, D.D., the Right Hon. Lord Knutsford, G.C M.G., Sir George B. Bruce, Sir Richmond Cotton, Sir F. Wyatt Truscott, Colonel Francis Haygarth, Mr. R. B. Martin, M P., and Messrs. R. B. D. Acland, H. C. Burdett, T. Christy, A. L. Cohen, J. G. Glover, M.D., J. H. Hale, C. J. Hare, M.D., A. G. Sandeman (Governor of the Bank of England), T. Wakley, F.R.C.S., Rev. J. Cundy, H. Hoskier, and H. N. Custance (Secretary).

Since the awards for the year 1895 were made additional subscriptions amounting to £16,000 have been placed in the hands of the Lord Mayor, and the Council were asked at the present meeting to sanction the distribution of the extra sum among the various hospitals and dispensaries that participated in the previous distribution, 5 per cent. of the total amount to be, as usual, reserved for the purchase of surgical appliances.

Letters of apology for absence were read from Sir Edmund Hay Currie, Mr. Thomas Bryant, Captain Palliser, Mr. Howard Potter, Bishop of London, Canon Ingram, Sir Savile Crossley, Mr. John Mackerel, Rev. C. J. Ridgeway, Lord Charles Bruce, Mr. Carr-Gomm, Mr. Richard Foster, Mr. Alfred Willett, Dr. Munro Gibson, and others.

Dr. Glover postponed his resolution for the appointment of a sub-committee to investigate the statistics of the out-patient departments at the various hospitals till another meeting.

Sir Sydney Waterlow, in rising to propose the adoption of the committee's report, said it was very gratifying to him, and he was sure they would all sympathise with him as chairman of the Distribution Committee, that the funds placed in the hands of the Lord Mayor made it necessary to call another meeting of the Council for the purpose of confirming a second report. This was the twenty-third year that a collection had been made, and never had a collection approached the sum that had been reached this year. They had always been struggling to reach £50,000, and now, owing to the energy of a large number of gentlemen, they were able to announce a collection of over £61,000. Immediately he learned that a further sum had been placed in the hands of the Lord Mayor he called a meeting of the Committee of Distribution, because the principle upon which the Fund had always been administered was to give the benefit of the money collected in the year to the sick poor of the metropolis without delay, and not to put any by. They had already distributed £45,000, and it was proposed to ask this meeting to confirm the second report, which recommended that a further sum of £15,850 should be divided amongst the several hospitals as set forth in the schedule. Before closing his remarks he wished to say that part of the proceedings of the Committee of Distribution had not been entered upon the report. The committee felt that they ought to express to Mr. Burdett their best thanks, and he hoped that the Council would include those thanks in any resolution they might pass to-day. It should also not be forgotten that the funds collected this year had been greatly increased by the very deep interest that the Lord Mayor had taken in the Fund ever since he had been in office, and he was sure that Sir Joseph Renals felt quite as strong a feeling of gratitude as he did that the public had recognised so largely his efforts to increase the Fund. He, therefore, begged to move that the second report of the Distribution Committee be approved.

Lord Knutsford, in seconding the resolution, which was unanimously carried, concurred in all that Sir Sydney Waterlow had said. He heartily joined in the praise and thanks which had been given to Mr. Burdett. No one could express how very much that Fund was indebted to him for its present position.

### Thanks for Services Rendered.

His Eminence Cardinal Vaughan then proposed for the Council's adoption a series of resolutions passed by the Committee of Distribution as follows:—

(1) That the thanks of this Council be given to Henry C. Burdett, Esq., for the great interest he has taken in the Metropolitan Hospital Sunday Fund for many years past, for the benefit that the Fund has derived from his advocacy of its claims in The Hospital newspaper, and by other means, but especially for his strenuous exertions and personal influence this year in obtaining subscriptions, raising the Fund to the unprecedented sum of over £60,000.

(2) That the thanks of the Council be given to B. I. Barnato, Esq., Daniel Marks, Esq., and their friends for the very handsome and generous contribution of £10,000 towards the Hospital Sunday Fund, thus assisting to raise the Fund to the unprecedented amount of over £60,000.

(3) That the thanks of the Council be conveyed to Messrs. Burdett and Harris, and Messrs. Pim, Vaughan, and Co. for their much-appreciated exertions in obtaining subscriptions amounting to £3,400 from members of the Stock Exchange.

He did not ask them to merely confirm these resolutions, but to adopt them as their own. He felt that it needed no words of his to commend these resolutions to the acceptance of the meeting. Mr. Burdett and the gentlemen whose services he had enlisted had put the metropolis under a deep obligation by the untiring zeal, the extraordinary exertions, and the great generosity bestowed upon this hospital charity. They asked for no thanks from men. They had acted from the noblest motives of charity and humanity, and they sought their recompense elsewhere and in the testimony of their own conscience. Still, it was fitting that the citizens at large should recognise conspicuous services rendered in this behalf; and therefore it was that this Council which represented them was most properly invited to confirm and make its own these resolutions expressive of hearty thanks and deep gratitude. Inasmuch as they were all deeply interested in the great work of ministering to the corporal necessities and wants of the poor in this great metropolis, they must feel all the deeper gratitude to these gentlemen who had helped to raise the sum for hospital distribution to the large amount recorded. They were bound to record their high appreciation of the time, influence, and money which had been placed at their disposal by these gentlemen for the benefit of the poor, so that the general public might understand how such services are appreciated. He felt also that to record their hearty thanks might serve as an indirect call to others to imitate the noble example set before them.

Mr. R. B. Martin, M.P., seconding the motion, thought that a vote of thanks ought particularly to be passed by this Council, because not only had the gift of money raised the income of the Fund, but it showed what an enormous amount of good could be done by individual efforts. These gentlemen had worked among their own circle of friends, and he thought the Council could not do less than record their names. The motion was heartily agreed to.

### Mr. Burdett's Reply.

Mr. Burdett said that it would be wrong for him not to acknowledge that the work he had done this year had been performed at considerable personal cost both of energy and time; but he felt that, however natural and however proper it might be for them to desire to personally thank him, still, the less the individual was brought forward on this occasion the better. Nevertheless, he very greatly appreciated these resolutions of thanks for personal service of which he was an apostle, and he thanked them from his heart. There was no way they could help the hospitals better than by showing what a measure of success the Fund could obtain. The unit of confidence was the Committee of Distribution; had it not been for them it would certainly have been quite impossible to raise the amount of money which they had raised this year. There were people who criticised the methods of distribution, and he himself in early years was among the number of the critics, but as he had grown older he had come to the conclusion that it would pass the wit of man to devise a scheme that would not lay itself open to the criticism of somebody. Knowing that the members of the committee are independent and not connected with any hospital, he thought he might say that they owed an immense debt to the committee, and that there was every confidence to be placed in their methods. Sir Sydney Waterlow was the acting spirit and head of the committee, and it was only right to regard that committee as the platform from which it had been possible to raise the Fund to £61,500.

With reference to the question of personal services, eight good friends, one of them a member of the Council—he had been forbidden to mention names—felt that they might have a little syndicate in the cause of charity amongst themselves, and he wished it to be remembered that in thanking him the Council were thanking those friends also. The resolution covered all those who had helped to raise the money. The figures representing the congregational collections showed an increase approaching £5,000, as compared with the previous year, and there was a deep debt owing to the clergy and ministers of all denominations. It would be invidious to single out names, but it was impossible to forget the special services which the Archdeacon of London had rendered this year, nor could they omit to thank especially the Archbishop of Canterbury, the Dean of Westminster, the Chief Rabbi, Canon Fleming, and Dr. Ridgeway.

There were a great number of people who, desiring to help hospitals, felt some difficulty in deciding what hospital to give to, and the Hospital Sunday Fund afforded a means to these people of placing their money in the hands of the Lord Mayor, knowing that it would be distributed among the hospitals according to their respective needs and merits. On this occasion he must emphasise the personal services of the present Lord Mayor, and he doubted very much the possibility of raising the money had it not been for his hearty co-operation. The Lord Mayor's secretary, too (Mr. Soulsby), had taken a great amount of trouble on behalf of the Fund, and had also devoted a large amount of time to it during the last twenty years. There had been several individual donations of £1,000—Sir Savile Crossley, Lord Iveagh, and Mr. J. B. Robinson. The Stock Exchange list included some 400 private donors and firms, and Mr. Barrato's list some 500 others. Altogether £20,000 had this year been sent to the Lord Mayor in donations alone by upwards of 1,200 citizens of London. From £27,000 in 1884 the total annual receipts of the Fund had gone up to £42,000 in 1890, and now, in 1895, to £61,500, and he believed that the Sunday Fund should never be less than £50,000 in any year. It was not impossible that in the future the sum reached might be £100,000. This was a very gratifying thought, and he maintained that the encouragement they had received this year proved that he might express reasonable confidence that the Sunday Fund would one day bring £100,000 to the coffers of the hospitals. It was to one man more than to any other, he believed, that they owed Hospital Sunday in London. He alluded to the late Dr. James Wakley (whose work had since been taken up by his brother, Mr. Thomas Wakley, to whom they were all so much indebted), and the success they had attained would be a matter of great satisfaction to him if he could know it. He thanked them once more for all their kindness to him that day.

Cordial Thanks to the Press.

Mr. Burdett then proposed—

That the grateful thanks of the Council be conveyed to the Press on behalf of the medical charities of London for the special support they have so cordially rendered to the effort to raise the Hospital Sunday Fund collections to the magnificent and unprecedented total which has been reached this year, and that a copy of this resolution, signed by the Lord Mayor, be forwarded by the secretary to the editor of each of the newspapers whose advocacy has been so fruitful in its results.

They owed much of their success to the Press. Had it not been for the kindness of Mr. W. Thomas, of the *Graphic*, in placing his artist, who alone could prepare the diagrams with accuracy, at his disposal, it would have been impossible for him to have brought out the special number of The Hospital with illustrations. Then, again, there was Mr. Arthur Walter, of the *Times*, who, setting aside all precedents, gave way on a point in the interests of the Hospital Sunday Fund, which he had never given way on before, by admitting blocks into the columns facing the leaders. He thought it was right he should mention this fact because it was a very materal contribution towards the results which had been attained. Other metropolitan editors [a complete list of them is given on page 67 of The Hospital for October 26th, 1895] had rendered material support, and, passing by his own paper, he could not sit down without specially thanking, and asking the Council to thank, the *Lancet*. It had devoted to the cause of Hospital Sunday an amount of space which he knew must have cost several hundred pounds, and it was to the *Lancet* that this year they owed a special "Hospital Sunday Supplement," which was circulated throughout the churches, and of which 125,000 copies were printed for gratuitous distribution. He asked the Council to pass this resolution, not in a formal manner, but with enthusiasm. The editors of the metropolitan press, though, as journalists, entrusted with the responsibility of conserving the interests of the empire, were individually and collectively citizens of London, and their recognition of this fact and the duties attaching to citizenship by their generous advocacy of the claims of the voluntary hospitals of London, would, he was confident, stimulate a multitude of other citizens to give of themselves in the cause of the medical charities.

Mr. Hoskier seconded, and the vote was heartily accorded.

The Chief Rabbi proposed that the cordial thanks of the Council be given to the Right Hon. Sir Joseph Renals, Bart., for his efforts in making the present year a record year of the Fund.

Canon Fleming seconded, and the motion was unanimously carried.

The Lord Mayor, in acknowledging the vote, said it was quite in the true fitness of things that almost the last meeting at which he should preside in this Mansion House should be one so deserving of the sympathy of every Englishman as the Hospital Sunday Fund. They all believed that this fund was gradually growing upon the public, and he hoped that ultimately even the figures suggested by his friend Mr. Burdett might be increased. If in the years to come he could help the Fund in his small way he should be only too happy to do so, and among the pleasant memories of the civic chair that he should leave behind him none would be more pleasant than the fact that during his year of office the Metropolitan Hospital Sunday Fund had risen to an amount it had never done before.

The proceedings then terminated.

